# Towards a Consensus for Dyslexia Practice: Findings of a Delphi Study on Assessment and Identification

**DOI:** 10.1002/dys.1800

**Published:** 2025-02-25

**Authors:** Caroline Holden, Philip Kirby, Margaret J. Snowling, Paul A. Thompson, Julia M. Carroll

**Affiliations:** ^1^ SpLD Assessment Standards Committee (SASC) Esher, Surrey UK; ^2^ King's College London London UK; ^3^ University of Oxford Oxford UK; ^4^ University of Warwick and University of Birmingham Coventry and Birmingham UK; ^5^ University of Birmingham Birmingham UK

**Keywords:** assessment, consensus, Delphi study, dyslexia, practice framework

## Abstract

This paper discusses the findings of a Delphi study in which dyslexia experts, including academics, specialist teachers, educational psychologists, and individuals with dyslexia, were asked for their agreement with a set of key statements about defining and identifying dyslexia: why it should be assessed and how and when this assessment should be conducted. Two rounds of survey responses provided a vehicle for moving towards consensus on how to assess for dyslexia. Forty‐two consensus statements were ultimately accepted. Findings suggested that assessment practice should take account of risks to the accurate identification of dyslexia. An assessment model, with guidelines for assessors, is presented, based on the Delphi's findings. This hypothesis‐testing model requires assessors to investigate and weigh up the factors most likely to result in an accurate assessment before reaching conclusions, assigning terminology, and making recommendations for intervention and management.

**Summary**

- Explores consensus in professional practice: why, when, and what to assess.
- Discusses the current assessment context for children with difficulties learning to read, spell, and write.
- Supports the ongoing use of the label dyslexia for persistent impairments in reading fluency and allied difficulties, such as spelling.
- Suggests an assessment framework for the identification of dyslexia.
- In the identification of dyslexia, highlights and discusses areas that require further research.

## 1 Introduction

How to identify dyslexia is a central question in dyslexia research and practice internationally (Wagner, Zirps, and Wood 2022; Snowling, Hulme, and Nation 2020; Poulsen, Juul, and Elbro 2023). It is also a key question in ongoing policy debates (Kirby 2020; Gearin et al. 2022). In the present journal, considering only the past decade, the question has been addressed from multiple perspectives: quality assurance issues in teacher‐based assessment of literacy difficulties (McMurray, O'Callaghan, and McVeigh 2018), diagnostic implications of the double deficit model (Harrison and Stewart 2019), the potential use of the simple view of reading in assessment practice (Sleeman et al. 2022), and inconsistencies in psychologists' identification methods (Sadusky et al. 2023).

This article builds on this work and related research that has surveyed the views of dyslexia assessors (Ryder and Norwich 2018) but offers a different approach to the question: it presents the results of a Delphi survey of dyslexia experts who were asked for their level of agreement with a series of statements about its nature and assessment (see Carroll et al., forthcoming, for more details on the study's methodology). A key benefit of the Delphi approach is that it is iterative, permitting refinement during the study to find areas of common agreement and disagreement. This study joins others, discussed below, which have recently sought to bring greater clarity to the dyslexia concept (Wagner and Lonigan 2023; Catts et al. 2024; Wagner et al. 2023; Wolf et al. 2024).

The present paper addresses two key questions: (1) To what extent does the revised dyslexia definition proposed by this Delphi study (see Table 1) move us towards a consensus for dyslexia assessment? (2) What implications does this Delphi study have for practitioners involved in assessing and identifying dyslexia?

**TABLE 1:** Delphi definition of dyslexia.

Dyslexia is a set of processing difficulties that affect the acquisition of reading and spelling (S8)
In dyslexia, some or all aspects of literacy attainment are weak in relation to age, standard teaching and instruction, and level of other attainments (S16)
Across all languages, difficulties in reading fluency and spelling are key markers of dyslexia (S4)
Dyslexic difficulties exist on a continuum and can be experienced to various degrees of severity (S19)
The nature and developmental trajectory of dyslexia depends on multiple genetic and environmental influences (S14)
Dyslexia can affect the acquisition of other skills, such as mathematics, reading comprehension or learning another language (S17)
The most commonly observed cognitive impairment in dyslexia is a difficulty in phonological processing (i.e., in phonological awareness, phonological processing speed or phonological memory). However, phonological difficulties do not fully explain the variability that is observed (S7)
Working memory, processing speed and orthographic skills can contribute to the impact of dyslexia (S31)
Dyslexia frequently co‐occurs with one or more other developmental difficulties, including developmental language disorder, dyscalculia, ADHD, and developmental coordination disorder (S18)

Arriving at a strong consensus answer to these questions is crucial for providing universal and equitable assessment for dyslexia. In the UK, as one example, there is presently little coherence in assessment methods within and between education sectors and levels and between the devolved administrations (England, Scotland, Wales, and Northern Ireland). Some English local authorities and county councils have stated that they do not recognise dyslexia or the need for specialist assessment (Henshaw 2018), while others are promoting ‘dyslexia‐friendly’ schools and policies (Cambridgeshire County Council 2019). In the UK Parliament, there is a well‐established All‐Party Parliamentary Group for Dyslexia and Specific Learning Difficulties, which meets regularly and invites interested bodies and stakeholding organisations to provide advice to policymakers, but there has been no significant legislation or policy directives regarding the assessment of dyslexia since 2019, when the Department for Education waived the age requirement for assessment evidence supporting applications for Disabled Students' Allowances (DSAs) in higher education (BDA 2019). The requirement for a post‐16 years assessment was removed on the basis that dyslexia (or another SpLD) should be considered a lifelong condition, so only one ‘diagnosis,’ made at any age, was required. In Scotland, by contrast, there is an agreed definition of dyslexia and a clear pathway to assessment (Education Scotland 2020). Such inconsistency is characteristic of other national contexts (see Maunsell 2020; Ontario Psychological Association 2018). Similarly, in the US, different dyslexia laws are adopted by different states, leading to unequal access to special education (Gearin et al. 2022).

A similar diversity of approaches is seen in psycho‐educational assessment. Specialist dyslexia practitioners may employ different rationales, evidence requirements, and assessment measures to identify a specific learning difficulty (see Andresen and Monsrud 2022; Sadusky et al. 2023). They may also apply different labels, including specific learning difficulty/difference/disability, specific literacy difficulties, reading disability/disorder, and, of course, dyslexia. These issues have implications both for the individuals assessed and for all those tasked with devising and implementing appropriate interventions and support. On seeking an assessment, parents, teachers, colleges, universities, and workplaces naturally wish to know whether an individual ‘is dyslexic’ and what can be done. While recent approaches include theoretical and pedagogic assessment through teaching (ATT), response to intervention (RTI), and MTSS (multi‐tiered systems of support) frameworks for the initial (and ongoing) assessment of, and interventions for, literacy difficulties, these typically do not incorporate a pathway for the identification of dyslexia (see Miciak and Fletcher 2020; Fuchs, Fuchs, and Compton 2004).

The implementation of continuous assessment frameworks such as ATT, RTI and MTSS challenges the concept of an early, one‐off diagnostic assessment that labels (or does not label) a child with a developmental difficulty ‘for life’, a practice that can entrench the unhelpful idea that the effects of developmental difficulties, such as dyslexia, are unchanging. The drive behind these models is one of ‘faultless instruction’, that is, failure to learn is viewed as a consequence of what is taught and cannot be attributed to any characteristics (e.g., ‘dyslexia’) of the student. Learner errors are seen as design flaws in instructional programmes and should lead to programmes of instruction being amended or refined.

Such frameworks in primary/elementary education tend to operate under the unproven assumption that interventions based on these practices will be universally successful, eliminating persisting difficulties and therefore the need for further assessment or a dyslexia label (Shanahan 2020). Similar claims have been made for the effectiveness of assistive technology and digital tools in obviating the impact of dyslexia (see Dawson et al. 2019).

The continual resurfacing of debates concerning the use of diagnostic labels, such as dyslexia, in the identification of literacy and learning difficulties has also highlighted the gatekeeper role of assessors regarding adjustments and resources, such as extra time in examinations, assistive technologies, and study support (SASC 2022).

One reason for the difficulty in achieving consensus around dyslexia's assessment is the continuing—and longstanding—debate over dyslexia's definition itself (Kirby and Snowling 2022). One risk of any consensus‐building process, such as the present Delphi (Carroll et al., forthcoming), is the danger of over‐inclusivity. The study authors sought to mitigate this risk by (1) setting a high bar for consensus (80% strong or moderate agreement with each statement) and (2) rewording and retesting statements that did not show initial consensus in the light of comments received. In a third phase held with a sub‐set of Delphi panel members, there was agreement that inclusive definitions offer fewer risks of excluding marginal individuals from support than under‐inclusive definitions.

Thus, although there were residual areas of controversy (specifically, the role of intellectual abilities in dyslexia, whether cut‐off criteria should be employed in assessment, and the extent to which reading impairment can be separated from dyslexia), the current Delphi study found continuing support for the term, a similar conclusion to that reached following discussions between researchers at the Florida Centre for Reading Research (Catts et al. 2024). The proposed definition (achieving 80% consensus) is shown in Table 1.

## 2 Methods

In 2021, the UK's SpLD Assessment Standards Committee (SASC) began a comprehensive consultation on current approaches to assessment in the UK, identifying where similarities and differences in practice existed. Based on this consultation (SASC 2022), the present Delphi study was conceived in 2023. The Delphi methodology has been recognised as an instructive way to gauge expert consensus where there is uncertainty about professional guidelines and is often employed in health‐related research (Hasson, Keeney, and McKenna 2000).

Ethical permission for the Delphi study was provided by Coventry University. The study recruited a panel of 71 dyslexia experts who gave informed consent to take part.

### 2.1 Participants

Dyslexia has been the subject of substantial interdisciplinary research; it was therefore important to ensure the panel represented a broad range of expertise. Moreover, given recent debates regarding dyslexia's nature and causes and best practice in assessment, it was important to include in the moderating group individuals leading two ongoing consultations at the British Psychological Society and SASC (JC and CH, respectively). Panel invitations were issued to representatives from the four UK nations (England, Scotland, Wales, and Northern Ireland; *n* = 32) working in education, psychology, and occupational support, and from national and international experts with an academic background from English‐speaking (Australia, Canada, UK, US) and non‐English‐speaking nations (Egypt, Finland, France, Germany, Hong Kong, Kuwait, Netherlands, Norway, Portugal, Sweden), some of whom were engaged in other national dyslexia organisations (e.g., European Dyslexia Association, SpELD Australia). The panel also included individuals with lived experience of dyslexia. Seventy‐one participants accepted and formed the expert panel. Of these, 58 provided responses to the survey in Round 1 and 57 in Round 2. Our panel was predominantly female (*n* = 41), with 17 males. The breakdown of panel members by country and discipline is shown in Table 2.

**TABLE 2:** Demographic details of first round panellists (*n* = 58).

Country	Profession	Stakeholder groups represented
England (73%) Scotland (9%) Wales (2%) Northern Ireland (2%) USA (2%) Europe (7%) Other (5%)	Academic (44%) Educational psychologist (9%) Specialist teacher and/or assessor (27%) Other (20%)	PATOSS (Professional Association of Teachers of Students with Specific Learning Difficulties) SASC (Specific Learning Difficulties Assessment Standards Committee) BDA (British Dyslexia Association) Dyslexia Action Working with Dyslexia Helen Arkell Dysguise

### 2.2 Survey

Fifty‐five statements were assembled by the moderating group (JC, CH, PK, MS) on five themes: (1) ‘The Nature and Causes of Dyslexia’; (2) ‘Experiences of Dyslexia’; (3) ‘Why and When to Assess’; (4) ‘What to Assess’; (5) ‘Identification Criteria’. These were taken from three sources: *The Science of Reading: A Handbook* (Snowling, Hulme, and Nation 2022), *The Dyslexia Debate* (Elliott and Grigorenko 2014), and a consultation paper (SASC 2022) being the output of a process involving over 400 assessment practitioners and academics. The expert panel responded to two successive rounds of statements, and the statements were modified in response to feedback. In total, 42 statements were agreed upon across the five themes (Table 3) and 11 statements were rejected (Table 4). Accepted statements are referred to in the text as S1, S2, S3, etc.

**TABLE 3:** Accepted statements.

Nature and causes of dyslexia
S1. A history of dyslexia in the family is a significant risk factor for dyslexia; however, the causes of dyslexia include multiple genetic and environmental factors
S2. Accounts of dyslexia that attribute dyslexia to a single cause such as weak phonology, or problems in working memory, do not account for individual variability or the highly overlapping nature of dyslexia with other disorders of learning
S3. There are differences in the manifestations of dyslexia, depending on how a language is written (orthography), its sound‐structure (phonology), grammar and morphology
S4. Across all languages, difficulties in reading fluency and spelling are key markers of dyslexia
S5. Cognitive processes that influence the skills required for literacy are likely to be impaired in dyslexia
S6. Orthographic processing refers to the ability to form and retrieve letters, letter sequences and spelling patterns, and is commonly impaired in dyslexia
S7. The most commonly observed cognitive issue in dyslexia is a difficulty in phonological processing (i.e. in phonological awareness, phonological processing speed or phonological memory). However, phonological difficulties do not fully explain the variability that is observed
S8. Dyslexia is a set of processing difficulties that affect the acquisition of reading and spelling
S9. The term developmental dyslexia distinguishes dyslexia with a childhood onset from cases of acquired dyslexia with a neurological cause (such as brain injury)
S10. Persistent and sometimes severe difficulties in word and non‐word decoding (reading accuracy) are typically observed in children with dyslexia learning to read and spell in English. Secondary consequences of dyslexia may include problems in reading comprehension and reduced reading experience that can impede growth of vocabulary and background knowledge
S11. While some older children and adults with dyslexia continue to experience word level reading problems, others mainly have difficulties in reading and writing fluency, and in spelling
S12. While there is suggestive evidence of an association between non‐right handedness (left or mixed handedness) and dyslexia, the information is not useful for identifying dyslexia
S13. Visual stress is a condition in which the visual system appears to be hypersensitive to high contrast regular patterns, including lines of black text against a white background. Visual stress is a separate condition to dyslexia but it can make it difficult to process text and hence may exacerbate reading difficulties
Experiences of dyslexia
S14. The nature and developmental trajectory of dyslexia depends on multiple genetic and environmental influences. The impact of dyslexia for any individual can change over time depending on circumstances and experiences
S15. Protective factors in dyslexia include early and sustained intervention, and good verbal, nonverbal and oral language skills
S16. In dyslexia, some or all aspects of literacy attainment are weak in relation to age, standard teaching and instruction, and level of other attainments
S17. Dyslexia can affect the acquisition of other skills, such as mathematics, reading comprehension or learning another language
S18. Dyslexia frequently co‐occurs with one or more other developmental difficulties, including developmental language disorder, dyscalculia, ADHD, and developmental coordination disorder
S19. Dyslexic difficulties exist on a continuum and can be experienced to various degrees of severity
S20. After intervention and appropriate support, reading and the associated difficulties of individuals with dyslexia may no longer be experienced as disabling, although they may remain challenging
S21. People with dyslexia may develop other skills as an adaptive process to compensate for literacy based difficulties. However, there is little evidence to support the idea that dyslexia confers advantages in, for example, creative or visual–spatial skills
Why and when to assess
S22. All individuals struggling with literacy require appropriate, targeted intervention, monitoring, and resources
S23. In the early years of reading instruction, the identification of needs of children with literacy learning difficulties should be prioritised over detailed diagnostic assessment. Detailed diagnostic assessment should not be a precondition for putting intervention in place
S24. Individuals with reading difficulties should be referred for specialist assessment if there is consistent lack of progress in reading or writing despite targeted assistance
S25. Good assessment and intervention practice embodies a hypothesis‐testing approach. Assessors should ask themselves what risk factors are at play, including risk of a longer‐term difficulty
S26. Ideally an assessment should seek input from other professionals in instances where there seem to be a range of co‐occurring difficulties (developmental, psychosocial, or medical)
S27. Assessment of dyslexia is required for many different purposes, for example, identification for research, for planning intervention, or for supporting individuals in the workplace. The content of the assessment needs to be aligned to its purpose
What to assess
S28. Multiple sources of information should be combined in assessment, including, for children, interview/questionnaires with parents or caregivers and liaison with the school, direct observation, and standardised age‐normed tests or criterion‐based assessments
S29. Useful indicators of the need to assess a school‐age child for possible dyslexia include: reference to results, where they exist in school, from standardised phonics checks; failure to meet age‐related targets in reading, writing, and spelling; discrepancies between literacy and language performance, and slow or no progress across 6–12 months of planned intervention
S30. To assess the level of severity or persistence of dyslexic difficulties, an examination of how the individual responds or has responded to interventions and support provides important information
S31. Working memory, processing speed and orthographic skills can contribute to the impact of dyslexia
S32. Assessing phonological processing and orthographic skills is important for identifying the impact of dyslexia on the individual concerned and to inform intervention
S33. Assessment of second or additional language learners requires an extra emphasis on knowledge and understanding of how a first language(s) (L1) might affect performance in tests of literacy attainment and cognitive processing in a second language (L2)
Identification criteria
S34. The following features may be indicative of dyslexia in the early years: (a) a family history of dyslexia; (b) slow acquisition of letter names and/or sounds; (c) difficulty blending and segmenting sounds; (d) slow naming speed; (e) particular difficulty reading nonwords, and non‐phonetic spelling errors
S35. Children who come to school with speech or language difficulties are at risk of literacy difficulties, including dyslexia
S36. In older children and adults, early and persisting literacy difficulties may have been missed or masked. It is important to investigate such histories to ascertain whether the current difficulties could be attributed to dyslexia
S37. When assessing older children and adults, information about whether they had difficulties in literacy in the early school years supports identification of dyslexia
S38. Adult assessments should aim to uncover factors that have limited an individual's literacy during their lifetime to make recommendations about intervention and support
S39. While qualitative observations and skilled professional judgements are important in the identification of dyslexia, standardised test results provide objectivity, consistency and reliability
S40. When an individual has generalised learning difficulties (intellectual disabilities) applying a dyslexia label may result in too narrow an approach to intervention
S41. Discrepancy between intellectual ability and literacy attainment is a useful indicator of a specific learning difficulty but is not sufficient for a diagnosis in and of itself
S42. Guidelines are needed so that assessments for dyslexia are consistent, but it is difficult to achieve consensus on criteria within these guidelines

**TABLE 4:** Rejected statements.

Nature and causes of dyslexia
R1. There is some evidence that preschool measures of brain structure predict variation in learning to read. However, it is not possible to use such findings to understand the risk of dyslexia in an individual child
R2. Sensorimotor deficits, including auditory processing problems, are associated with dyslexia
R3. Poor visual attention is a causal factor in dyslexia
R4. Neurodiversity is a better term to use than dyslexia
Experiences of dyslexia
R5. Many people with dyslexia experience difficulties with oral communication skills
What to assess
R6. In young children, tests of word/nonword reading and spelling, alongside tests of phoneme awareness, phonological short‐term memory and orthographic processing skills should be sufficient for screening for dyslexia
Identification criteria
R7. Younger children (to 7–9 years) who, after 6–12 months sustained and monitored intervention for literacy needs, show a significant narrowing of an age‐related attainment gap in reading, writing or spelling skills are unlikely to be at risk of dyslexia
R8. A clear difference between stronger oral language skills and weaker written language skills can be used to establish evidence for dyslexia
R9. Dyslexia can be present in an individual regardless of additional problems (e.g. difficulties with vision or hearing) but the label should not be used in the presence of intellectual disability or where low levels of attainment can be attributed to lack of exposure to literacy
R10. In the identification of dyslexia in children, cut‐off criteria, for example, scores of at least 1SD below the mean for age in one or more standardised tests of literacy skill, are important in establishing evidence for the persisting difficulties characteristic of dyslexia. They provide guidance and prevent over‐identification
R11. It is normally possible to distinguish between an individual with dyslexia and an individual with poor reading skill

*Note:* Refer Table 3 caption.

Our focus in this paper is on dyslexia assessment, that is, the results of themes (3) and (4) and accepted statements 22–33. We draw on the broader study to situate these findings (see Carroll et al., forthcoming). Statements have been thematised in the general discussion for clarity, so they are not necessarily discussed in their original numerical order.

### 2.3 Procedure

The first phase of the survey was emailed in May 2023. Panellists rated each question once on a five‐point Likert scale–‘Strongly Disagree’, ‘Somewhat Disagree’, ‘Neither Agree nor Disagree’, ‘Somewhat Agree’, ‘Strongly Agree’–or could select ‘No Opinion/Do Not Know’. Each statement provided the opportunity for the expert to make comments regarding the appropriateness of language and phrasing and to provide citations to support their position. The statements were distributed as an electronic survey using the Qualtrics platform.

Fifty‐eight panellists (out of 71 invitees) participated in Round 1; 57 participated in Round 2. Once data collection was complete, the independent analyst/data controller [PT] collated the percentages of responses in each category for each statement into an anonymised report for the moderators [JC, CH, MS, PK] and into individual reports for each panel member. The moderators scrutinised the collated comments and reworded, amalgamated, or removed statements as necessary.

Round 1 contained 55 statements, of which 27 were accepted as achieving consensus (defined as at least 80% combined agreement in the ‘Strongly Agree’ and ‘Somewhat Agree’ categories), 7 were removed due to high levels of disagreement, and 21 were modified for review in Round 2. Following Round 2, 18 further items were accepted, and four items were removed where consensus was not obtained. A subset of panel participants was invited to a further meeting in January 2024 to discuss the findings and remaining issues. Anonymised data, reports and R scripts can be found on the Open Science Framework (https://osf.io/vhxgf/).

## 3 Findings and Discussion

### 3.1 On Supporting All Individuals With Literacy Difficulties

All individuals struggling with literacy require appropriate, targeted intervention, monitoring, and resources, regardless of socio‐economic situation (S22).

This Delphi study began by seeking consensus on a foundational principle: is assessment necessary for literacy difficulties (whatever term is used to describe these)? There was almost unanimous agreement that all individuals struggling with literacy require (and are entitled to) appropriate intervention, monitoring, and resources. Illustrative comments from participants include: “I don't think anyone could dispute this”; “I cannot agree with this strongly enough”. As noted, this seemingly straightforward statement nevertheless goes unpracticed, or inconsistently so, by many education authorities worldwide (Maunsell 2020).

The importance of providing appropriate support for all individuals was also reflected in statements about approaches to assessment in primary school‐aged children.

In the early years of reading instruction, the identification of needs of children with literacy learning difficulties should be prioritised over detailed diagnostic assessment. Detailed diagnostic assessment should not be a precondition for putting intervention in place (S23).

This priority was considered especially important between the ages of 5 and 8 years. Participants also recognised the importance of close observation of any difficulties and of how the child responds to intervention as central to understanding and supporting literacy acquisition as a foundation for learning.

### 3.2 On Employing a Probabilistic ‘at Risk’ Framework

As the following three statements illustrate, there was general support for an ‘at risk’ or ‘vulnerabilities’ approach to assessment in the early years of schooling, with follow‐up monitoring and, potentially, adjustment of classroom and other support strategies before the application of a label.

Children who come to school with speech or language difficulties are at risk of literacy difficulties, including dyslexia (S35).

Individuals with reading difficulties should be referred for specialist assessment if there is consistent lack of progress in reading or writing despite targeted assistance (S24).

Useful indicators of the need to assess a school‐age child for possible dyslexia include reference to results, where they exist in school, from standardised phonics checks; failure to meet age‐related targets in reading, writing, and spelling; discrepancies between literacy and language performance; and slow or no progress across 6–12 months of planned intervention (S29).

Although it was acknowledged that detailed diagnostic assessment and labelling should not be a precondition for putting intervention in place, most respondents nevertheless felt that individuals with reading difficulties should be referred for specialist assessment if there is a consistent lack of progress in reading or writing, despite targeted assistance: “Ideally there would be intervention prior to any diagnostic assessment with the diagnostic assessment taking place because the intervention has not worked”.

There was also consensus around indicators that are useful when judging the optimal time point to further assess a school‐age child for dyslexia: “Intervention should obviously begin as early as possible in a child's educational career as soon as risks are evident, but the ‘critical stage’ [authors' rewording] for diagnostic assessment is KS2 (ages 8 and 9 in the UK) [when] there is enough historical evidence and evidence of RTI to make an informed decision”. There was some scepticism expressed regarding the usefulness of the UK's Phonics Check (after 2 years of reading instruction) and re‐check as a progress measure, alongside concerns that monitoring interventions for 6–12 months may constitute too long a ‘wait’ for fuller assessment. It was also noted that “Taking an ‘at risk of developing dyslexia’ approach is helpful at this young age. However, there will always be exceptions, particularly for those with the severest difficulties”.

### 3.3 Evaluating Persistence

To assess the level of severity or persistence of dyslexic difficulties, an examination of how the individual responds or has responded to interventions and support provides important information (S30).

This statement implies a model of assessment in which intervention is provided according to needs, and response to intervention is closely monitored to inform possible later assessment. This aligns with England's *Special educational needs and disability code of practice* *: 0–25 years* (DfE and DoH 2015). In the US, the MTSS framework provides a more formal approach.

Despite consensus on the importance of evaluating response to intervention, there was less consensus regarding the notion, tested in the first round of the Delphi survey, that a significant narrowing of the age‐related attainment gap in reading, writing, or spelling skills, following intervention in children aged 7–9 years, means that the child is unlikely to be at risk of dyslexia—in other words, a positive response to intervention by a child in this age range should not rule out the possibility of dyslexia. Most of this hesitancy relates to doubts about the quality or appropriateness of intervention or a feeling that some children may be responding to intervention, but only by expending an unusual amount of effort in the process, a sign that underlying cognitive processing difficulties may still act as a hindrance to progress.

Relatedly, there were queries from participants about what qualified as an acceptable intervention and as an acceptable response to that intervention. For example: “I agree [with S30], although of course we also must then include in our analysis an evaluation of the quality of the support and interventions, which usually proves quite problematic”; and “I think we need guidance on what is meant by a response, for how long they [the child] would access the intervention, and what would be expected as not responding”. There was concern about curriculum appropriateness, especially a narrow or exclusive focus on systematic synthetic phonics and the assessment thereof: “Failure to meet age‐related targets may be more reflective of the teaching methods used and the age‐related targets set… than a likelihood of dyslexia”. In their recent paper revisiting the definition of dyslexia, Catts et al. (2024, 8), also note that “more attention needs to be given to how to account for the quality and quantity of instruction in diagnosing and identifying dyslexia.”

Some participants also felt that it would be important to assess earlier in instances where a child appeared to be experiencing multiple or severe difficulties and in situations where difficulties appeared to have been overlooked and few or no interventions offered. A participant noted that “I don't think prior targeted assistance should be a prerequisite for assessment. Schools are busy places, and some difficulties can be missed”.

Two statements were relevant to investigating the persistence of difficulties in older children and adults:

When assessing older children and adults, information about whether they had difficulties in literacy in the early school years supports identification of dyslexia (S37).

In older children and adults, early and persisting literacy difficulties may have been missed or masked. It is important to investigate such histories to ascertain whether the current difficulties could be attributed to dyslexia (S36).

There was considerable support in the survey responses for the importance of using the learning history of the individual assessed as an element of converging evidence towards identification, if not as “definitive diagnostic criteria”. However, the second statement above offers an important qualifying position. This statement encourages assessors to consider difficulties emerging “as the conceptual pitch and pace of work increases”, “where compensatory strategies no longer work, or when they are too difficult or tiring to sustain” or where it is simply not possible to gain such information.

### 3.4 How Should Assessments Be Approached?

Good assessment and intervention practice embodies a hypothesis‐testing approach. Assessors should ask themselves what risk factors are at play, including risk of a longer‐term difficulty (S25).

There was consensus that good assessment practice should embody a hypothesis‐testing approach that is flexible and considers the possibility of longer‐term and/or co‐occurring difficulties for the individual: “It should not be acceptable to examine a single domain and feel that the job is done”; “The background information might inform the tests that you decide to use, but we would emphasise the importance of an open mind”.

### 3.5 On Gaining Information From Multiple Sources

Gaining information from multiple sources is key to considering and assessing hypotheses about the nature of an individual's difficulties. Two consensus statements addressed this directly.

Multiple sources of information should be combined in assessment, including, for children, interviews/questionnaires with parents or carers and liaison with the school, direct observation, and standardised age‐normed tests or criterion‐based assessments (S28).

While qualitative observations and skilled professional judgements are important in the identification of dyslexia, standardised test results provide objectivity, consistency, and reliability (S39).

There was consensus that standardised tests are a vital component in the assessor's toolkit when used in conjunction with other factors such as parental discussion and direct observation: “This is essential”; “Crucial”. It was stressed that the combination of these elements of assessment provides the best likelihood of correct identification: “We need converging evidence to support full consideration and promote accuracy”; “I agree with the statement as long as all three components qualitative observations, skilled professional judgements, and standardised tests‐are used and the findings synthesised”. Some agreement with this statement was caveated with the assessor's expertise in psychometrics: “Yes–if the right tests are chosen and administered/interpreted appropriately”.

These multiple sources may involve other professionals working with an individual.

Ideally, an assessment should seek input from other professionals in instances where there seem to be a range of co‐occurring difficulties (developmental, psychosocial, or medical) (S26).

There was agreement that an assessment should seek input from other professionals in instances where there seem to be a range of co‐occurring difficulties, but there was also acknowledgement that this may require too much resource: “I don't think we need a multidisciplinary team to consider dyslexia, but the child would benefit from a multidisciplinary approach so we get to consider the whole picture”; “A joined‐up approach is vital”; “Many assessors work in isolation. A network to facilitate this would be very helpful”; and “Absolutely essential for pre‐16s and highly advisable for those older (if the adult is willing).” At the same time, it was noted that “Resources makes this counsel of perfection hard to achieve in many [education] authorities”.

### 3.6 On Dyslexia and L2 Learners

Assessment of second or additional language learners requires an extra emphasis on knowledge and understanding of how a first language(s) (L1) might affect performance in tests of literacy attainment and cognitive processing in a second language (L2) (S33).

Delphi participants agreed that the assessment of second or additional language learners requires appreciation of how proficiency in a first language(s) (L1) might affect performance in a second language (L2) when administering tests of literacy attainment and cognitive processing. While some guidance is available (e.g., in the UK context, SASC 2019), participants felt that this was an important area where further training and professional development is required: “I think we need tighter guidelines on the assessment of EAL [English as an Additional Language] students. And we should admit that accurate assessment (e.g., of phonology and literacy attainments) is simply not possible in cases where English skills are still developing. It is also important to compare oral vocabulary knowledge in English and literacy attainments”; “There needs to be more training for assessors on this”; “A good assessor would always be considering how socio‐cultural, personality, and linguistic factors are impacting performance”. Arguably, to gain a better understanding of the needs of dyslexic learners who speak minority languages, it is important to turn to multilingual contexts where guidelines for assessment and intervention have been developed (e.g., Cárdenas‐Hagan 2020; Nag 2017).

### 3.7 What Should Be Included in an Assessment?

Our consensus statements established that assessment should use a hypothesis‐testing approach and combine various sources of information to address a multiple‐deficit conceptualisation of dyslexia. To test hypotheses, the contents of the assessment should be aligned with those potential hypotheses.

Assessing phonological processing and orthographic skills is important for identifying the impact of dyslexia on the individual concerned and to inform intervention (S32).

Working memory, processing speed and orthographic skills can contribute to the impact of dyslexia (S31).

There was strong agreement that any diagnostic assessment should consider the role of phonological skills, orthographic skills, processing speed, and working memory. The wording of the Delphi statements focused on assessing these factors for the purpose of establishing impact on attainment, rather than as causes of dyslexia. However, participants noted issues surrounding interpretation: “I would see these as specific learning needs in their own right that impact more than just literacy”; “I agree very strongly [with S31], but…how do we differentiate slow processing that affects everything from slow processing that only affects literacy?” “You need to know more than this [S32], but getting a sense of which literacy ‘building blocks’ are vulnerable gives you a good sense of the degree of strain on a person's literacy processing in downstream, higher‐level skills, including reading fluency and comprehension”.

Regarding the purpose of including, in assessment, tests of intellectual abilities, the Delphi study reached marginal consensus on the following two statements:

Discrepancy between intellectual ability and literacy attainment is a useful indicator of a specific learning difficulty but is not sufficient for a diagnosis in and of itself (S41).

When an individual has generalised learning difficulties (intellectual disabilities), applying a dyslexia label may result in too narrow an approach to intervention (S40).

In the companion Delphi paper (Carroll et al., forthcoming), the issue of discrepancy in the identification of dyslexia is discussed in the context of evolving definitions of dyslexia. The lack of complete agreement in widely used definitions and in the theoretical literature mirrors many of the responses to the survey statements. While many comments are supportive of the first statement above (S41), some raise the issue of the specificity of a learning difficulty. “If we can't identify strengths across the profile, then can we diagnose (dyslexia)?” Some suggest that the testing of intellectual abilities should be to establish ‘potential’ rather than ‘actual’ performance and so garner reasonable adjustments to account for such disparities, where there is such evidence. Others feel that the testing of intellectual abilities can establish unexpectedness in a profile. One person suggests that “Intellectual ability is correlated with listening comprehension, so a discrepancy between intellectual ability and literacy attainment will be correlated with a discrepancy between reading comprehension and listening comprehension.” On the other hand, several comments suggest that intellectual abilities do not predict response to intervention.

On the issue of applying the dyslexia label when there is evidence of intellectual disability, there was concern that this practice could endanger the specificity in dyslexia, that is, the notion of unexpectedness; it is also the case that few standardised measures are sensitive enough at the lower end to determine such discrepancy. There is also a concern that interventions may need to be different in this group, for example, delivered “in smaller steps”, with “more review”. Some respondents did take an opposite view, suggesting that the dyslexia label is less stigmatising than others, that those with “generalised learning difficulties” “are not a homogeneous group”, and that “dyslexia‐friendly teaching and learning strategies are good for all”.

### 3.8 On the Use of Guidelines for Assessment

Assessment of dyslexia is required for many different purposes, for example, identification for research, for planning intervention, or for supporting individuals in the workplace. The content of the assessment needs to be aligned to its purpose (S27).

There was consensus for the statement (S27) that dyslexia assessments are required for multiple purposes—from support during education to workplace requirements and participation in research studies (see Bartlett, Moody, and Kindersley 2010) and that assessments should be aligned to these purposes. The important role of professional judgement was recognised as existing within a process of reaching agreement on common approaches, led by guidance.

Guidelines are needed so that assessments for dyslexia are consistent, but it is difficult to achieve consensus on criteria within these guidelines (S42).

There was consensus regarding the need for consistency, but mixed comments on adhering to set guidelines for assessment—perhaps, in part, because the statement was imperfectly constructed. There was strong agreement with the need for guidelines, although flexibility within those guidelines for “skilled judgement” was seen as crucial: “Guidelines are very important and should be as comparable as possible across countries”. Some participants felt the notion of guidelines did not go far enough: “Guidelines are not strong enough. There needs to be set, clear criteria”; “We must aim for a definition and set of criteria for dyslexia that are simple and universal and based on what is measurable”. Reference was made to regularly updated clinical guidelines for assessment that exist in countries such as Germany, which have been found helpful (see Galuschka and Schulte‐Körne 2016). Notably, according to these, a reading disorder can only be diagnosed if reading skills are below average for age.

There was also acknowledgement that guidelines might be difficult to produce and that different criteria might be required for different age groups: “If one thinks that the criteria should vary, for instance between child and adult, then good criteria should include a specification of how they vary”. Generally, participants who wanted clear, measurable criteria also tended to think that these would be straightforward to achieve.

## 4 General Discussion

The present Delphi study posed two key questions: (1) To what extent does the revised dyslexia definition proposed by this Delphi study move us towards a consensus for dyslexia assessment? (2) What implications does this Delphi study have for practitioners involved in assessing and identifying dyslexia?

Before discussing how successfully these aims were achieved, it is important to note that queries were raised by a minority of respondents as to whether the label ‘dyslexia’ was useful at all (for further discussion, see Elliott 2020), with some preferring the more general term ‘literacy difficulty’ given concerns that, for younger children, identification of literacy‐based needs might be best considered as an ongoing, corroborative assessment process, gaining information from multiple sources, including RTI. Despite this, the present consensus was that the term ‘dyslexia’ remains useful. Here, our findings align with the recommendations of the Rose (2009) review on the assessment of dyslexia and specific literacy difficulties, as well as recent academic opinion that has revisited the concept (Wagner et al. 2023; Catts et al. 2024). Similarly, this Delphi study acknowledges that there is a high co‐occurrence between dyslexia and other specific learning difficulties (that may affect the impact of dyslexia). However, this study goes further than Rose (2009) in supporting a definition of dyslexia associated not only with phonological processing difficulties, but with a complex aetiology involving multiple potential factors or ‘risks’ (Catts and Petscher 2022; Catts et al. 2024; McGrath, Peterson, and Pennington 2020). Future research should seek to define the most probable factors associated with developmental dyslexic trajectories more precisely.

An important implication of this multifactorial framework is that a hypothesis‐testing approach should underpin assessment practice. This approach must be based on a recognition that the probable causes of dyslexia involve complex interactions between biological (genes, brains) and environmental factors. Clearly, it is critical that this approach be evidence‐based. For example, further research is required to differentiate causal risk factors for dyslexia from secondary factors affecting prognosis, some of which may be downstream effects of poor reading, such as difficulties in orthographic processing. Arguably, early impairment of phonological skills would necessarily affect the development of orthographic representations and hence lead to orthographic deficits (Burt 2006; Deacon, Benere, and Castles 2012).

Additionally, some constructs such as ‘processing speed’ would benefit from much greater scrutiny as to how exactly they should be both conceptualised and assessed given that they involve multiple brain processing systems. Any aspect of processing, phonological, visual or other, is likely to involve interactions between some or all of these areas. Some tests attempt to measure specific elements of processing, for example, phonological processing speed (the rate at which a person takes in, makes sense of, and responds to auditory information) or visual processing speed (the response time needed to correctly search and/or reach for a visual stimulus), but there is still much to learn about neurobiological bases and the impact of processing ‘inefficiencies’.

Alongside phonological processing and orthographic skills, other factors such as working memory, oral language skill, attention control, processing speed, and certain environmental factors may play a part in the way dyslexia manifests for some individuals (Catts et al. 2024). However, this does not mean that all these factors need always to be assessed. In assessment practice, it therefore seems important to move away from overconfidently asserting the assumed *causes* of an individual's dyslexia towards assessment of the impact of the key factors likely to be involved.

Given the present state of the science and the complex interplay of genetic, biological, cognitive, and environmental factors on development, it is impossible for assessors to confirm the causes of dyslexia in any individual. Indeed, guidelines for the identification of developmental learning disorders, such as ICD‐11, echo a cautious approach (WHO 2024b). While individuals with dyslexia may typically show impairments in certain psychological processes, assessors can only establish potential cognitive vulnerabilities, the effects of which are observed in tests of reading, spelling and writing skills and which could also affect other aspects of life and learning.

One rationale for conducting the present study was the view from assessors that the recommendations of Rose (2009) lack clear guidelines for assessment, particularly in relation to the role of IQ and of cut‐off criteria for the identification of dyslexia. As many participants commented, there is a danger that adopting very strict and inflexible cut‐off points, in the absence of considering other variables and factors, may exclude some individuals from access to much‐needed intervention. The expert panel did not achieve complete consensus on this issue. In the Delphi follow‐up meeting, it was noted by most attending panellists that cut‐off criteria are desirable in order to take action, but that, simultaneously, such criteria will exclude some children and adults with reading and spelling difficulties who require support.

Similarly, regarding the much‐debated role of the assessment of intellectual abilities in the identification of dyslexia, there remains disagreement (Di Folco et al. 2022). The ICD‐11 (WHO 2024b) definition endorses the validity of the discrepancy definition, while DSM‐5 (APA 2017) is moot on this point. As argued elsewhere (Snowling, Hulme, and Nation 2020), the likelihood that reading difficulties will be accompanied by co‐occurring conditions is inevitably raised at lower IQ levels, because the wider range of difficulties will themselves affect the measurement of IQ (e.g., spatial difficulties in ‘dyspraxia’ affecting performance IQ, or comprehension deficits in children with language difficulties who typically have lower verbal IQ).

We suggest that a notable discrepancy between intellectual ability and literacy attainment, for those individuals where this is evident, can help suggest that literacy‐based difficulties are unexpected, but that this is not the only relevant measure of unexpectedness. Other measures could include persistence of difficulties despite standard instruction and/or additional support and comparisons with performance in school progress tests for mathematics, science, arts and technology. In the ICD‐11 World Health Organisation definition, although the term developmental learning disorder is the preferred term rather than dyslexia, the advice is that a diagnosis should consider “various sources of evidence regarding the child's capacity for learning outside the formal testing situation” (WHO 2024a). Using multi‐scale IQ or cognitive abilities assessment measures may also provide additional valuable information that can influence parent and teacher expectations, help to identify co‐occurring difficulties and compensatory resources, and guide intervention methods, allowing assessors to arrive at a more rounded and comprehensive picture of the individual's strengths and weaknesses.

While a strength of the Delphi study is that it involved practitioners working in the field of dyslexia who could offer insights based on longstanding, reflective practice, this also illuminated areas of disagreement between academic theory and assessment practice. Achieving a balance between clear, measurable criteria in the assessment of dyslexia and the inevitable ambiguities involved in skilled professional judgement is critical. Good practice in assessment is not merely ticking boxes as to whether an individual fulfils certain criteria; it involves weighing up multiple factors and considering how each may contribute to the profile of that individual. We propose that professional judgements should involve a transparent process, observing guidelines and exercising professional judgement, of weighing up information and evidence from all three components of assessment: background information, qualitative observations, and standardised test results, mindful of risks introduced by expectancy biases and errors of measurement.

We argue that this hypothesis‐testing approach guides most of the consensus statements on why, when and what to assess. If one is embodying a hypothesis‐testing approach, then it is appropriate: to gain information from multiple sources and aim to combine it; to encourage well‐founded intervention without necessarily the need for a diagnosis; to consider carefully the purpose of the assessment; and to assess a range of cognitive skills. Such observations sit with the limitations of all assessment tools. Not all tests are well‐standardised (and some norms are dated), some tests are not suited to the population being assessed (e.g., because of cultural or linguistic factors), and relatively few tests are co‐normed. Furthermore, many tests offer only a snapshot picture of the individual's abilities; test profiles are not stable over time, difficulties may not fully emerge in the individual until later, and there are many factors that can affect test performance on the day (Ryder and Norwich 2018).

Notwithstanding this, and in line with Rose (2009), there was strong agreement in this Delphi study that a consistent lack of progress in reading, spelling, or writing is a significant factor to be taken into account when assessing need. There was enthusiasm for the consideration of responses to intervention, where information is available, as providing valuable information about persisting difficulties that could be described as dyslexia. However, there were also dissenting responses, noting difficulties in operationalising RTI. Indeed, despite early promise, the RTI framework is now acknowledged to have limitations and arguably may provide only as much information as do measures of the severity of difficulties (e.g., Peng et al. 2020).

A critical point is the need for awareness of the social and political context of assessment. A recent Delphi study of parents of dyslexic children identified two perceived barriers to diagnosis: inadequate training for teachers and, relatedly, insufficient funding for dyslexia in schools (Harding et al. 2023). There remains a ‘postcode lottery’ in educational provision (Hutchinson 2021), meaning that, where assessment and labelling are regarded (or required) as the only route to intervention, many children can be left unsupported. A recent study from the LSE (Campbell 2023: iv) on differential labelling depending on social economic group concluded that:

Children living in more deprived areas are more likely to be categorised with less specific, more common SEND [special educational needs and disability] ‘types’–‘Speech, Language and Communication Needs;’ ‘Moderate Learning Difficulties;’ and ‘Social, Emotional and Mental Health Difficulties.’ Children in more affluent areas have higher chances than those in poorer areas of being diagnosed with less prevalent, more precisely defined conditions that involve agencies outside of the school in ascription: ‘Autistic Spectrum Disorder;’ ‘Specific Learning Difficulties;’ ‘Physical Disabilities;’ ‘Severe Learning Difficulties;’ ‘Hearing/Visual/Multisensory Impairments'; ‘Profound and Multiple Learning Difficulties.’ These patterns hold, controlling for children's own FSM [free school meals] eligibility, ethnicity, home language, and LA [local authority] of residence.

Special education provision is also stratified by socio‐economic group, gender, and ethnicity. In England, for example, boys make up nearly two‐thirds of pupils with SEND support, and 37.5% of pupils with SEND support are eligible for free school meals (a proxy for socio‐economic disadvantage), compared to 23.8% of pupils in all schools (Gov.uk 2023). More than a quarter of five‐year‐olds in England did not meet the expected standard for literacy in 2022/23, with higher rates in deprived areas of the country (Cabrera, Gomez, and Franklin 2024). Strand and Lindorff (2018) point to inequalities and disproportionalities in the experience of provision among different ethnic groups.

Thus, approaches to dyslexia assessment have policy implications. This Delphi study confirmed consensus that, in the very early years of instruction, it should be possible to establish which children are at risk of literacy and related learning delay and respond with ongoing intervention. For those children who show persisting difficulties with learning to read and spell (and/or in mathematical learning), there should be pathways to more comprehensive assessment of dyslexia or, if the assessment evidence suggests this, another specific difficulty such as dyscalculia or a developmental language disorder (DLD).

Although the Delphi study did not examine intervention as such, progressive, intervention‐focused assessment that is mindful of the dangers of persistence and accumulating risks supports the need to intervene (if not necessarily label) at an early stage of literacy acquisition. This model can minimise the risk of a downward spiral of deteriorating literacy skills and accompanying psychosocial problems and benefit all children struggling to read, spell and write.

## 5 Limitations

It is reasonable to ask whether the outcomes of this study would have been different if the author group (who devised and moderated the statements) or the invited Delphi panellists (whose views were surveyed) had been weighted less towards the opinions and perspectives of researchers and assessment professionals and more towards dyslexic individuals. We acknowledge that any Delphi study turns on the constitution of the panel. However, although the authors invited a number of panellists who were known to have self‐reported dyslexia, the expert panel was not asked to declare if they themselves identified as dyslexic. The primary purpose of this study was not to compare the views of those who identified as dyslexic with those who did not, but rather to involve a diverse sample of people with considerable professional and/or personal experience of researching, assessing, teaching and working with individuals with dyslexia. To our knowledge, the current study is unique in involving both assessment practitioners and academics. Indeed, another Delphi study (Gearin et al. 2024) remarks particularly on the previous absence of the voice of practitioners and policy makers in the International Dyslexia Association's (IDA) definition of dyslexia, thereby inadvertently creating implementation challenges for school practice.

Nevertheless, it would certainly now be possible to test the outcomes of this study using the agreed consensus statements with a different panel to seek validation of the findings. Indeed, following an earlier Delphi study of developmental language disorder, several professional bodies replicated the process (e.g., Kristoffersen et al. 2021), and others tested the uptake of its recommendations (e.g., Gallagher et al. 2023).

### 5.1 Moving Forwards: A Model for Planning Dyslexia Assessment

**FIGURE 1:**
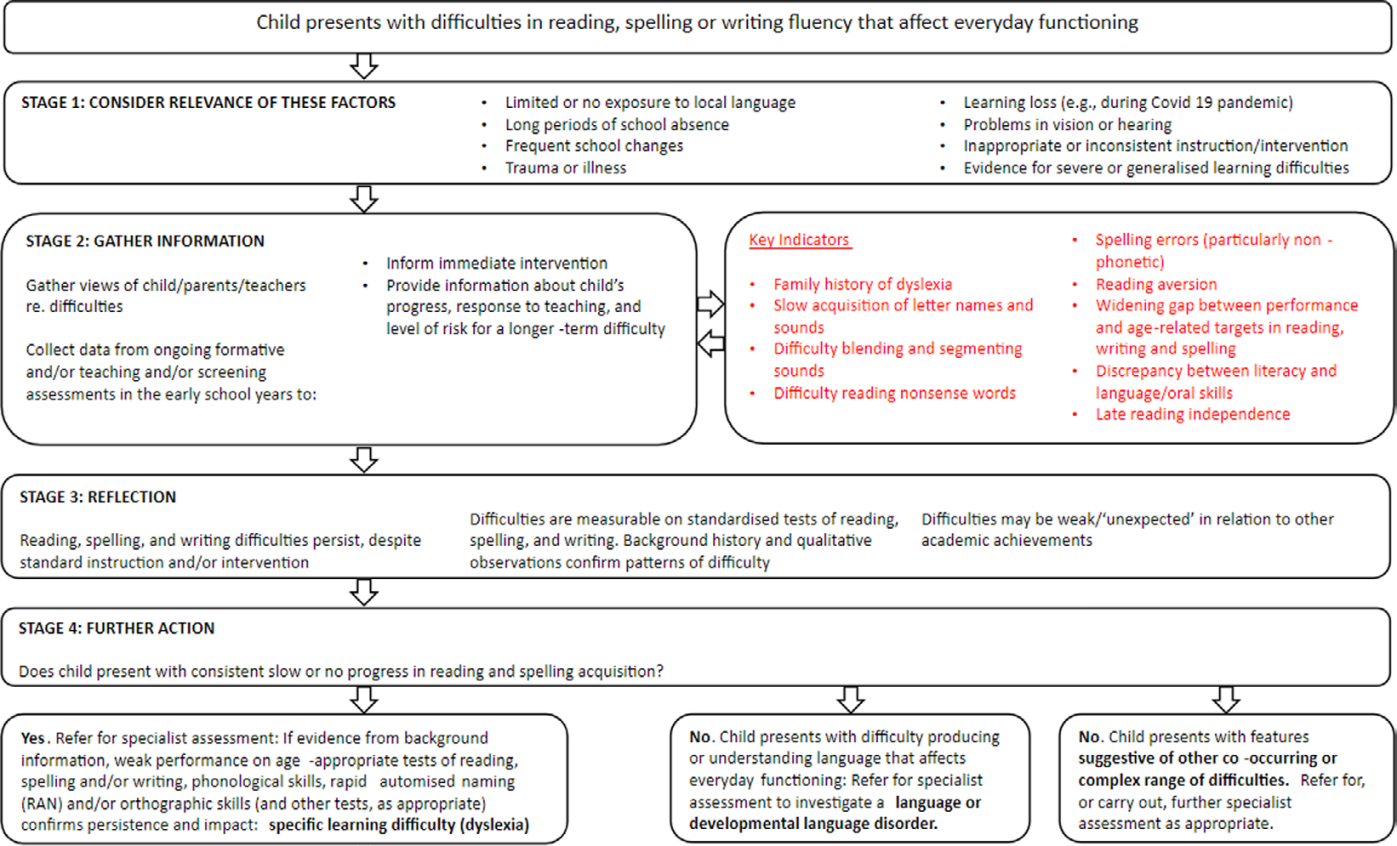
Hypothesis‐testing model for dyslexia assessment—child.

**FIGURE 2:**
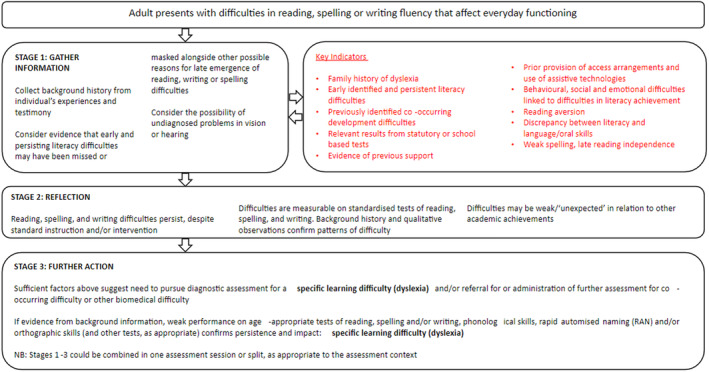
Hypothesis‐testing model for dyslexia assessment—adult.

The results of the Delphi study highlight the complexity involved in assessing dyslexia and the need to draw upon multiple sources of information: background information, standardised test results, and qualitative observations. Skilled professional judgement (mindful of expectancy biases) must be balanced against clear measurable criteria (taking account of measurement error). Importantly, professionals need to be aware of the socioeconomic disparities in access to assessment/diagnosis. A hypothesis‐testing approach moves thinking forward in terms of the assessment of dyslexia, recognising the strengths and limitations of both professional judgement and standardised assessments. So, while prescriptive guidelines have not been identified, this study has achieved what it set out to do regarding models for assessment of children (see Figure 1) and adults (see Figure 2).

We propose a hypothesis‐testing model involving factors that we suggest increase the probability of the accurate identification of dyslexia. This model encapsulates the key areas of consensus captured by the Delphi study and is intended to provide a useful, at‐a‐glance summary for academics, practitioners, and policymakers of key areas of agreement regarding why, when and what to assess to achieve the greatest likelihood of the accurate identification of dyslexia.

Each model includes a multi‐stage process considering information from different sources. Notably, the childhood model suggests that finding difficulties in reading, spelling, or writing is only the first step in moving towards a dyslexia assessment. Before a full diagnostic assessment is considered, we propose consideration of alternative explanations and an information‐gathering phase that includes some information as to the child's response to classroom intervention. This is in line with the Delphi statements indicating that in young children, assessment of need should be prioritised over diagnosis. With respect to the adult model, a consideration of alternative explanations is more complex. A careful history of an individual's literacy progress throughout school should be taken. Nonetheless, the key indicators are similar to those in childhood. An elaborated version of this model, intended to provide more precise guidance to assessment practitioners, is available online (www.sasc.org.uk).

## 6 Conclusion

The findings of this Delphi study support the ongoing need to identify persistent literacy difficulties, of which the cardinal feature is an impairment of reading fluency, as ‘dyslexia’. The study highlighted the risk of abandoning all descriptive labels in the field of specific learning difficulties/disorders for accessing intervention, and an alternative term, ‘neurodiversity’, was not considered preferable by panel participants in the context of statements written to test consensus around a definition and assessment criteria for dyslexia.

The adoption by some of a neurodiversity framework when writing about dyslexia stems largely from a genuine wish to acknowledge an individual's strengths and to avoid the potential social and emotional problems associated with negative labelling and deficit models of classification. However, an unintended and potentially harmful consequence of this approach can also lead to “stereotyped forms of promoting dyslexia as a gift that comes with specific gifts or advantageous traits that characterise it” (Odegard and Dye 2024, 147).

In an assessment context, and from a disability rights perspective, individuals still need to seek identification to ensure necessary support and accommodations in education and the workplace. This identification necessarily involves examining problems in reading and writing that affect everyday functioning and present challenges for the individual concerned. Skilled assessment practitioners are acutely aware of the need to avoid stereotypes, both positive and negative, and to take account of the feelings, perspectives and experiences of the individuals they assess. They also play a key role in identifying the most effective interventions and strategies for the individual concerned in the context in which they are learning, working, or living. In this Delphi study, there was strong support for retaining the label dyslexia and for assessment guidelines based on an agreed definition (see Carroll et al., forthcoming).

When considering implications for practitioners involved in assessing and identifying dyslexia, there is never likely to exist a simple ‘recipe’ to guide its identification. However, it is possible to offer a set of principles, guided by the consensus statements of this study, which could usefully underpin assessment practice, particularly for children and young people, but are equally applicable to adults in further or higher education and in the workplace.

## Conflicts of Interest

The authors declare no conflicts of interest.

## Data Availability

The data that support the findings of this study are openly available in Open Science Framework at https://osf.io/vhxgf/.

## References

[dys1800-bib-0002] American Psychiatric Association (APA) . 2017. Diagnostic and Statistical Manual of Mental Disorders. Washington, DC: American Psychiatric Association.

[dys1800-bib-0003] Andresen, A. , and M.‐B. Monsrud . 2022. “Assessment of Dyslexia – Why, When, and With What?” Scandinavian Journal of Educational Research 66, no. 6: 1063–1075.

[dys1800-bib-0004] Bartlett, D. , S. Moody , and K. Kindersley , eds. 2010. Dyslexia in the Workplace: An Introductory Guide. Oxford: Wiley‐Blackwell.

[dys1800-bib-0005] British Dyslexia Association (BDA) . 2019. “DfE Changes to the Requirements for Assessment Practising Certificates (APC).” https://www.bdadyslexia.org.uk/news/dfe‐changes‐to‐the‐requirements‐for‐assessment‐practising‐certificates‐apc.

[dys1800-bib-0006] Burt, J. 2006. “What Is Orthographic Processing Skill and How Does It Relate to Word Identification in Reading?” Journal of Research in Reading 29, no. 4: 400–417.

[dys1800-bib-0007] Cabrera, K. , R. Gomez , and J. Franklin . 2024. Early Literacy Matters: Economic Impact and Regional Disparities in England. London, UK: Pro Bono Economics. https://www.probonoeconomics.com/early‐literacy‐matters.

[dys1800-bib-0008] Cambridgeshire County Council . 2019. “Cambridgeshire Literacy Difficulties/Dyslexia Guidance.”

[dys1800-bib-0009] Campbell, T. 2023. “Inequalities in Provision for Primary Children With Special Educational Needs and/or Disabilities (SEND) by Local Area Deprivation.” *CASE Paper 213*. London School of Economics.

[dys1800-bib-0001] Carroll, J. M. , C. Holden , P. Kirby , M. J. Snowling , and P. A. Thompson . Forthcoming. “Contemporary Concepts of Dyslexia: A Delphi Study.” Journal of Child Psychiatry and Psychology.

[dys1800-bib-0010] Cárdenas‐Hagan, E. 2020. Literacy Foundations for English Learners: A Comprehensive Guide to Evidence‐Based Instruction. Baltimore, MD: Brookes.

[dys1800-bib-0011] Catts, H. , and Y. Petscher . 2022. “A Cumulative Risk and Resilience Model of Dyslexia.” Journal of Learning Disabilities 55, no. 3: 171–184. 34365842 10.1177/00222194211037062

[dys1800-bib-0012] Catts, H. , N. Terry , C. Lonigan , et al. 2024. “Revisiting the Definition of Dyslexia.” Annals of Dyslexia 74: 1–21. 38194056 10.1007/s11881-023-00295-3 PMC12063701

[dys1800-bib-0013] Dawson, K. , P. Antonenko , H. Lane , and J. Zhu . 2019. “Assistive Technologies to Support Students With Dyslexia.” Teaching Exceptional Children 51, no. 3: 226–239.

[dys1800-bib-0014] Deacon, S. H. , J. Benere , and A. Castles . 2012. “Chicken or Egg? Untangling the Relationship Between Orthographic Processing Skill and Reading Accuracy.” Cognition 122, no. 1: 110–117. 22030120 10.1016/j.cognition.2011.09.003

[dys1800-bib-0015] Department for Education (DfE) and Department of Health (DoH) . 2015. Special Educational Needs and Disability Code of Practice: 0 to 25 Years. London: Department for Education.

[dys1800-bib-0016] Di Folco, C. , A. Guez , H. Peyre , and F. Ramus . 2022. “Epidemiology of Reading Disability: A Comparison of DSM‐5 and ICD‐11 Criteria.” Scientific Studies of Reading 26, no. 4: 337–355.

[dys1800-bib-0017] Elliott, J. 2020. “It's Time to Be Scientific About Dyslexia.” Reading Research Quarterly 55, no. S1: S61–S75.

[dys1800-bib-0018] Elliott, J. , and E. L. Grigorenko . 2014. The Dyslexia Debate. Cambridge: Cambridge University Press.

[dys1800-bib-0019] Fuchs, D. , L. Fuchs , and D. Compton . 2004. “Identifying Reading Disabilities by Responsiveness‐To‐Instruction: Specifying Measures and Criteria.” Learning Disability Quarterly 27, no. 4: 186–256.

[dys1800-bib-0020] Gallagher, A. , S. Finne , R. Dolan , and E. Dunphy . 2023. “Exploring the Uptake of CATALISE Recommendations From the Perspective of Speech and Language Therapists Working in the Irish Context: A Qualitative Online Survey.” Advances in Communication and Swallowing 26, no. 1: 13–23.

[dys1800-bib-0021] Galuschka, K. , and G. Schulte‐Körne . 2016. “The Diagnosis and Treatment of Reading and/or Spelling Disorders in Children and Adolescents.” Deutsches Ärzteblatt International 113, no. 16: 279–286. 27159142 10.3238/arztebl.2016.0279 PMC4985523

[dys1800-bib-0022] Gearin, B. , Y. Petscher , C. Stanley , N. Nelson , and H. Fien . 2022. “Document Analysis of State Dyslexia Legislation Suggests Likely Heterogenous Effects on Student and School Outcomes.” Learning Disability Quarterly 45, no. 4: 267–279.

[dys1800-bib-0023] Gearin, B. , J. Turtura , K. Anderson , et al. 2024. “An Interdisciplinary Perspective on the Strengths and Weaknesses of the International Dyslexia Association Definition of Dyslexia.” Annals of Dyslexia 74: 337–354. 10.1007/s11881-024-00310-1. 38867023

[dys1800-bib-0024] Gov.uk . 2023. “Academic Year 2022/23: Special Educational Needs in England.” https://explore‐education‐statistics.service.gov.uk/find‐statistics/special‐educational‐needs‐in‐england.

[dys1800-bib-0025] Harding, S. , M. Chauhan‐Sims , E. Oxley , and H. Nash . 2023. “A Delphi Study Exploring the Barriers to Dyslexia Diagnosis and Support: A Parent's Perspective.” Dyslexia 29, no. 3: 162–178. 37313635 10.1002/dys.1743

[dys1800-bib-0026] Harrison, A. , and M. Stewart . 2019. “Diagnostic Implications of the Double Deficit Model for Young Adolescents With Dyslexia.” Dyslexia 25, no. 4: 345–359. 31697024 10.1002/dys.1638

[dys1800-bib-0027] Hasson, F. , S. Keeney , and H. McKenna . 2000. “Research Guidelines for the Delphi Survey Technique.” Journal of Advanced Nursing 32, no. 4: 1008–1015. 11095242

[dys1800-bib-0028] Henshaw, C. 2018. “Council Attacked for Saying Dyslexia ‘Questionable’.” *TES*. https://www.tes.com/news/council‐attacked‐saying‐dyslexia‐questionable.

[dys1800-bib-0029] Hutchinson, J. 2021. Identifying Pupils With Special Educational Needs and Disabilities. London: Education Policy Institute.

[dys1800-bib-0030] Kirby, P. 2020. “Dyslexia Debated, Then and Now: A Historical Perspective on the Dyslexia Debate.” Oxford Review of Education 49, no. 4: 472–486. 10.1080/03054985.2020.1747418 PMC7455059 32939102

[dys1800-bib-0031] Kirby, P. , and M. J. Snowling . 2022. Dyslexia: A History. Montreal: McGill‐Queen's University Press. 36730539

[dys1800-bib-0032] Kristoffersen, K. , E. Kristian , A.‐L. Rygvold , et al. 2021. “Terminologi for Vansker Med Spark Hos Barn og Unge – En Konsensusstudie.” Norsk Tidsskrift for Logopedi 3: 6–23.

[dys1800-bib-0033] Maunsell, M. 2020. “Dyslexia in a Global Context: A Cross‐Linguistic, Cross‐Cultural Perspective.” Latin American Journal of Content and Language Integrated Learning 13, no. 1: 92–113.

[dys1800-bib-0034] McGrath, L. , R. Peterson , and B. Pennington . 2020. “The Multiple Deficit Model: Progress, Problems, and Prospects.” Scientific Studies of Reading 24, no. 1: 7–13. 32440085 10.1080/10888438.2019.1706180 PMC7241589

[dys1800-bib-0035] McMurray, S. , P. O'Callaghan , and C. McVeigh . 2018. “Quality Assurance Issues in the Teacher‐Based Assessment of Students With Literacy Difficulties for Examination Access Arrangements.” Dyslexia 24, no. 1: 3–16. 29314436 10.1002/dys.1576

[dys1800-bib-0036] Miciak, J. , and J. Fletcher . 2020. “The Critical Role of Instructional Response for Identifying Dyslexia and Other Learning Disabilities.” Journal of Learning Disabilities 53, no. 5: 343–353. 32075514 10.1177/0022219420906801 PMC7560958

[dys1800-bib-0037] Nag, S. 2017. Assessment of Literacy and Foundational Learning in Developing Countries. London: Department for International Development.

[dys1800-bib-0038] Odegard, T. , and M. Dye . 2024. “The Gift of Dyslexia: What Is the Harm in It?” Annals of Dyslexia 74: 143–157. 38877328 10.1007/s11881-024-00308-9

[dys1800-bib-0039] Ontario Psychological Association . 2018. Guidelines for Diagnosis and Assessment of Children, Adolescents, and Adults With Learning Disabilities: Consensus Statement and Supporting Documents. Ontario: Ontario Psychological Association.

[dys1800-bib-0040] Peng, P. , D. Fuchs , L. Fuchs , et al. 2020. “Is “Response/No Response” Too Simple a Notion for RTI Frameworks? Exploring Multiple Response Types With Latent Profile Analysis.” Journal of Learning Disabilities 53, no. 6: 454–468. 32623947 10.1177/0022219420931818 PMC7537763

[dys1800-bib-0041] Poulsen, M. , H. Juul , and C. Elbro . 2023. “A National Test of Dyslexia.” Annals of Dyslexia 73: 337–355. 37418132 10.1007/s11881-023-00285-5 PMC10522507

[dys1800-bib-0042] Rose, J. 2009. Identifying and Teaching Children and Young People With Dyslexia and Literacy Difficulties. London: Department for Children, Schools and Families.

[dys1800-bib-0043] Ryder, D. , and B. Norwich . 2018. “What's in a Name? Perspectives of Dyslexia Assessors Working With Students in the UK Higher Education Sector.” Dyslexia 24: 109–127. 29577523 10.1002/dys.1582

[dys1800-bib-0044] Sadusky, A. , E. Berger , A. Reupert , and N. Freeman . 2023. “Methods Used by Psychologists for Identifying Dyslexia: A Systematic Review.” Dyslexia 28, no. 2: 132–148. 10.1002/dys.1706 34931397

[dys1800-bib-0045] Scotland, E. 2020. Making Sense Programme: Final Report. Edinburgh: Scottish Government.

[dys1800-bib-0046] Shanahan, T. 2020. “What Constitutes a Science of Reading?” Reading Research Quarterly 55, no. S1: S235–S247.

[dys1800-bib-0047] Sleeman, M. , J. Everatt , A. Arrow , and A. Denston . 2022. “The Identification and Classification of Struggling Readers Based on the Simple View of Reading.” Dyslexia 28, no. 3: 256–275. 35766340 10.1002/dys.1719 PMC9542070

[dys1800-bib-0048] Snowling, M. J. , C. Hulme , and K. Nation . 2020. “Defining and Understanding Dyslexia: Past, Present and Future.” Oxford Review of Education 46, no. 4: 501–513. 32939103 10.1080/03054985.2020.1765756 PMC7455053

[dys1800-bib-0049] Snowling, M. J. , C. Hulme , and K. Nation , eds. 2022. The Science of Reading: A Handbook. 2nd ed. Hoboken, NJ: Wiley‐Blackwell.

[dys1800-bib-0050] Specific Learning Difficulties Assessment Standards Committee (SASC) . 2019. “Guidance on the Assessment of Individuals for Whom English is an Additional Language (EAL) and/or Where There is a Complex Linguistic History.” Esher, Surrey. https://sasc.org.uk/media/rcpl2mk0/eal‐assessment‐sasc‐guidance‐nov‐2019.pdf.

[dys1800-bib-0051] Specific Learning Difficulties Assessment Standards Committee (SASC) . 2022. Consultation Paper on the Identification of and Effective Intervention for Literacy Difficulties in Children and Adults. Esher, UK: Implications for the Assessment of Dyslexia. http://www.sasc.org.uk/media/ucnmqy3d/sasc‐consultation‐full‐paper‐april‐2022.docx.

[dys1800-bib-0052] Strand, S. , and A. Lindorff . 2018. Ethnic Disproportionality in the Identification of Special Educational Needs (SEN) in England: Extent, Causes and Consequences. Oxford and London: University of Oxford and Department for Education.

[dys1800-bib-0053] Wagner, R. , and C. Lonigan . 2023. “Early Identification of Children With Dyslexia: Variables Differentially Predict Poor Reading Versus Unexpected Poor Reading.” Reading Research Quarterly 58, no. 2: 188–202. 37448987 10.1002/rrq.480 PMC10338016

[dys1800-bib-0054] Wagner, R. , J. Moxley , C. Schatschneider , and F. Zirps . 2023. “A Bayesian Probabilistic Framework for Identification of Individuals With Dyslexia.” Scientific Studies of Reading 27, no. 1: 67–81. 36685047 10.1080/10888438.2022.2118057 PMC9851422

[dys1800-bib-0055] Wagner, R. , F. Zirps , and S. Wood . 2022. “Developmental Dyslexia.” In The Science of Reading, edited by M. J. Snowling , C. Hulme , and K. Nation , 2nd ed., 416–438. Oxford: Wiley‐Blackwell.

[dys1800-bib-0056] Wolf, M. , R. Gotlieb , S. Kim , et al. 2024. “Towards a Dynamic, Comprehensive Conceptualization of Dyslexia.” Annals of Dyslexia 74: 303–324. 10.1007/s11881-023-00297-1. 38217783 PMC11413046

[dys1800-bib-0057] World Health Organization (WHO) . 2024a. “6A03 Developmental Learning Disorder.”

[dys1800-bib-0058] World Health Organization (WHO) . 2024b. “6A03.0 Developmental Learning Disorder With Impairment in Reading.” https://icd.who.int/browse/2024‐01/mms/en#1008636089.

